# Assessment of dynamic cerebral blood flow changes during cognitive tasks in patients with post-COVID-19 syndrome

**DOI:** 10.1093/braincomms/fcag036

**Published:** 2026-02-10

**Authors:** Dieter F Kutz, René Garbsch, Frank C Mooren, Boris Schmitz, Claudia Voelcker-Rehage

**Affiliations:** Department of Neuromotor and Exercise, University of Münster, Münster 48149, Germany; Otto Creutzfeldt Center for Cognitive and Behavioral Neuroscience, University of Münster, 48149 Münster, Germany; Department of Rehabilitation Sciences, Faculty of Health, University of Witten/Herdecke, 58455 Witten, Germany; DRV Clinic Königsfeld, Center for Medical Rehabilitation, 58256 Ennepetal, Germany; Department of Rehabilitation Sciences, Faculty of Health, University of Witten/Herdecke, 58455 Witten, Germany; DRV Clinic Königsfeld, Center for Medical Rehabilitation, 58256 Ennepetal, Germany; Department of Rehabilitation Sciences, Faculty of Health, University of Witten/Herdecke, 58455 Witten, Germany; Department of Neuromotor and Exercise, University of Münster, Münster 48149, Germany; Otto Creutzfeldt Center for Cognitive and Behavioral Neuroscience, University of Münster, 48149 Münster, Germany; JICE, Joint Institute for Individualisation in a Changing Environment, University of Münster and Bielefeld University, 48149 Münster,Germany

**Keywords:** SARS-CoV-2, cognitive impairment, functional near-infrared spectroscopy (fNIRS), sample entropy, moment-to-moment variability

## Abstract

The objective of this study was to quantify the variability of cortical blood flow during cognitive load as an indicator of disease-related changes in cerebral capillary blood flow intermittency in patients with post-COVID-19 syndrome. The regulation of cerebral blood flow in the dorsolateral prefrontal cortex under cognitive load was examined using high-resolution functional near-infrared spectroscopy in 36 subjects including 12 patients with post-COVID-19 syndrome and two control groups [12 coronary artery disease patients matched for age and 12 young healthy individuals (CTRL)]. To induce cognitive load, a Flanker task and an N-back task were employed. The structure of temporal variability of local blood flow regulation was assessed using sample entropy at 17 channels spanning both brain hemispheres. The spatial variability of the regional blood flow pattern was evaluated using the coefficient of variation (CV) from sample entropies across all channels. Results revealed a notable discrepancy in that patients with post-COVID-19 syndrome exhibited reduced temporal variability (lower sample entropy) but elevated spatial variability (higher CV) in comparison to coronary artery disease patients during cognitive load (*P* = 0.02). In the N-back task, the spatial variability increased from healthy individuals to coronary artery disease patients to patients with post-COVID-19 syndrome and was associated with longer reaction time and with lower accuracy. The results confirmed that dynamic cerebral blood flow is altered in patients with post-COVID-19 syndrome, which may be related to fatigue during cognitive tasks. Sample entropy and CV values represent different aspects of blood flow regulation fluctuation. Their simultaneous analysis enabled a meaningful distinction between groups suggesting disease-related changes in brain haemodynamic. The presented method is therefore suitable for describing current states of cortical blood flow regulation and for documenting intervention results in patients with post-COVID-19 syndrome or patients with similar symptoms (e.g. myalgic encephalomyelitis/chronic fatigue syndrome).

## Teaser

Kutz *et al.* found that post-COVID-19 syndrome patients had altered cerebral blood flow, which was quantified using sample entropy (for temporal variability) and its coefficient of variation (CV) (for spatial variability). The results showed that Post-COVID-19 Syndrome patients had significantly reduced temporal complexity but increased spatial irregularity, which correlated with poorer cognitive performance.

## Graphical Abstract

**Graphical Abstract:**
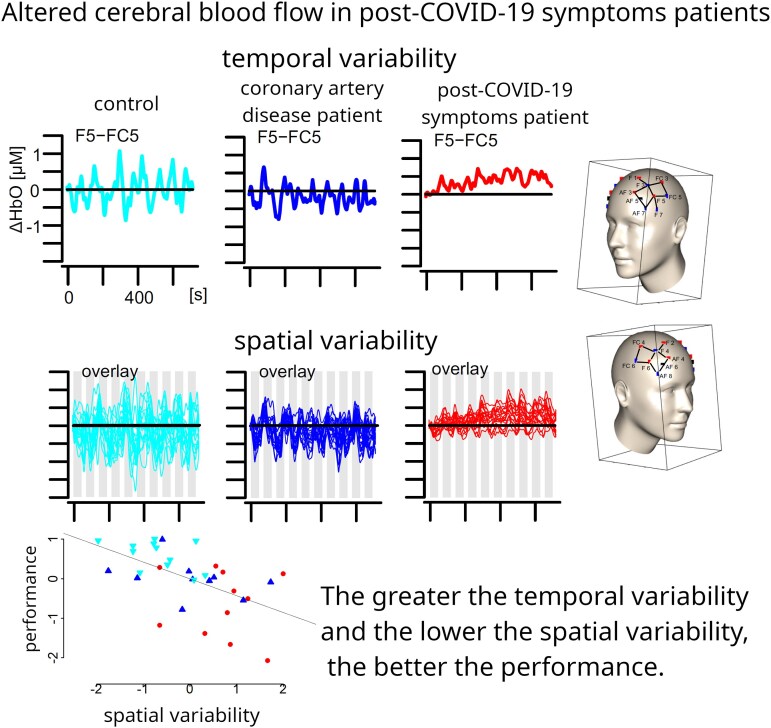

## Introduction

The post-COVID-19 syndrome (PCS) is characterized by persistent symptoms lasting beyond 12 weeks following an acute SARS-CoV2 infection or by emerging new symptoms within that period.^1,2^ Current guidelines for diagnosing PCS rely on various criteria, including ongoing complaints from the acute phase of illness, newly developed limitations or symptoms, or deterioration of pre-existing medical conditions.^1,3^ Symptoms vary widely in severity between affected individuals, ranging from mild disturbances to severe restrictions in daily life.^3,4^ Common symptoms include chronic fatigue, diminished physical capacity, muscle weakness and pain, respiratory difficulties, cognitive impairment (memory loss, concentration issues, sometimes referred to as ‘brain fog’), as well as psychological stress indicative of post-traumatic reaction.^3-6^ Although the aetiology of PCS remains poorly understood, potential causes comprise endothelial damage,^7,8^ a cytokine storm impacting multiple organs and cellular structures including mitochondria, along with alterations in central and peripheral blood flow regulation.^2,9-11^

The brain is an energetically demanding tissue, which requires a constant fine-tuning of its energy supply^12^ through mitochondria and accordingly supporting blood flow. Emerging evidence suggests that mitochondrial dysfunction and therewith energy deficiencies may contribute to the persistence and diversity of PCS symptoms.^13^ The brain attempts to compensate for these energy deficiencies in neurons and glial cells by altering blood flow and therewith O2 supply. Accordingly, studies using 18F-FDG-PET revealed significant glucose hypometabolism in various higher cortical areas, including the frontal and parietal cortex, insula, and para-hippocampal and fusiform gyrus in patients with an acute COVID-19 infection indicative of impaired mitochondrial function.^14-17^ Over the course of 5 months, a declining tendency of this hypometabolism has been noted, although restricted frontal clusters remained.^15^ Moreover, a correlation between the extent of hypometabolism and the degree of cognitive dysfunction in acute patients with cognitive impairment was found.^14,15^ Further, side-stream dark field microscopic analyses of the sublingual microvasculature suggested that endothelial damage may be the basis of the disturbed haemodynamic profile in PCS.^18,19^ Endothelial damage, in turn, can lead to a considerable and enduring reduction in vessel density, predominantly affecting the smallest capillaries^18,19^ and probably leading to a disturbed haemodynamic profile. This finding suggests that cortical capillaries, which typically account for ∼40% of the total vascular density,^20^ may also be affected. In this regard, changes in grey matter perfusion have been demonstrated,^21^ especially in bilateral frontal cortices and temporal cortices.^22^ These changes were associated with a significant oxygen deficiency in the brains of PCS patients compared with those of control subjects.^23^ The structural brain abnormalities have been shown to correlate with functional changes such as altered white matter functional connectivity and impairments in episodic memory, general cognition, attention and verbal fluency.^24^ Further, PCS patients have been characterized by hypoconnectivity between left and right para-hippocampal areas and between bilateral orbitofrontal and cerebellar areas.^25^

Incorporating combined electroencephalography (EEG) and functional near-infrared spectroscopy (fNIRS) substantiated these findings, reporting a disturbed haemodynamic profile in the prefrontal cortex.^26^ Blood flow is regulated by the smooth muscle cells of the arteries and arterioles supplying the brain, as well as by the contractile capillary pericytes.^12^ It has been shown that capillary pericytes respond to local energy demands by expanding the capillary diameter, granting pericytes a niche role in regulating blood flow compared to the more slowly responding arterioles.^12^ This means that, when energy requirements increase, a brief increase in local blood flow is induced in order to supply oxygen and glucose and remove metabolic waste products with an overall intermittent blood flow change in arterioles and capillaries.^20^ Of note, COVD-19 has been demonstrated to result in pericyte-mediated capillary constriction, leading to a reduction in cerebral cortical blood flow.^12^

Furthermore, disruptions of the endothelium may lead to an increase in capillary transit time heterogeneity (CTH) reported in healthy participants as well as in PCS patients.^27,28^ This is typically accompanied by a reduction in oxygen supply to the tissue, which is compensated by an increased local blood flow.^27-29^ The increased microcirculatory flow is enabled by an augmented influx of blood into the capillary network and an elevated number of shunt capillaries within a network.^27,28^ Endothelial alterations induced by COVID-19 may thus temporarily raise CTH beyond critical levels resulting in a significant drop in tissue oxygen concentration potentially causing symptoms such as memory impairment, cognitive fatigue or muscle pain.^29^ For instance, it has been demonstrated that increased blood perfusion in the grey matter and hippocampus of PCS patients is associated with an approximately ten times higher risk of daytime sleepiness.^21^

Given the altered CTH,^29^ local and regional blood flow regulation would be affected. This could lead to a reduction in intermittent blood flow regulation. The temporal structure of this variability can be characterized using sample entropy,^30-32^ a method for assessing the complexity of time-series signals, similar to heart rate variability.^33,34^ A higher sample entropy indicates a more pronounced intermittency of the regulatory process, whereas a lower sample entropy value suggests a more rigid regulation (and more self-similar signal) that is less able to adapt to local changes on demand. These changes might also be depicted in the temporal complexity of blood flow fluctuations in the in the dorsolateral prefrontal cortex as indicated by a lower sample entropy. Furthermore, regional differences in CTH might result in a more spatially irregular pattern of regional blood flow,^27-29^ which is characterized by different sample entropy across distinct measurement channels of a brain region (e.g. frontal cortex). This spatial distribution of the regional blood flow pattern can be characterized by determining the coefficient of variation (CV) from the sample entropies across all channels.

This study aimed to investigate changes in the temporal patterns of cerebral blood flow of PCS patients. We hypothesized that the temporal complexity of blood flow fluctuations in the dorsolateral prefrontal cortex would be altered in PCS patients, as reflected by differences in sample entropy between PCS patients and healthy controls. Specifically, we predicted that PCS patients would exhibit (i) lower sample entropy, indicating decreased intermittency of blood flow and (ii) a more widespread spatial distribution of diminished intermittency. Secondly, we hypothesized that PCS patients would demonstrate lower behavioural performance in cognitive tasks compared to healthy controls and that the behavioural differences would be linked to blood flow patterns.

We expect that analysing the temporal complexity of the cerebral blood flow would be a valuable tool for elucidating the complex interplay between cortical blood flow regulation and PCS. A reduced signal complexity might be an indicator of altered blood flow regulation due to an increased heterogeneity of CTH. This knowledge might inform the development and testing of personalized therapeutic strategies and treatment plans including targeted neuromodulation strategies, ultimately paving the way for more effective management of PCS and related disorders (e.g. ME/CFS^35,36^).

## Materials and methods

### Ethics committee

The study was approved by the local ethical review committee (Ethikkommission Universität Witten/Herdecke; reference number 159/2021 and 115/2020 for PCS and CAD patients, respectively) and the ethics committee of the Faculty of Psychology at the University of Münster (Germany) (reference 2023-17-CVR-FA) for CTRL participants. Written informed consent was obtained from all participants prior to research participation. The present study conformed to the Declaration of Helsinki.

### Participants

An exploratory cross-sectional study with PCS patients with cognitive impairment from the Clinic Königsfeld, Center for Medical Rehabilitation (Ennepetal, Germany) was performed between Mai and September 2023. In order to differentiate between disease-related changes and changes related to age, PCS patients were compared with control groups consisting of age- and sex-matched individuals with coronary artery disease (CAD) also undergoing medical rehabilitation as well as younger, healthy participants (CTRL). All patients were recruited while participating in an inpatient rehabilitation program described in detail elsewhere.^2^ Hence, the CAD group is defined as a no-treatment concurrent control group in accordance with ICH-E10 guidelines (Step 5) of the European Medicines Agency.^37^ Participants were selected from the same population as the test group, as they also undergo medical rehabilitation and exhibited comparable comorbidities to those of the PCS patients, with the exception of PCS-specific symptoms. Thus, the CAD group can be considered largely analogous to the PCS group with respect to central confounding variables that may exert an influence on the outcome. In addition, a group of healthy young participants (CTRL) was studied in the Institute of Sport and Exercise Sciences, University of Münster (Germany) between October and November 2023.

#### PCS patients

Inclusion criteria were a history of (at least one) COVID-19 infection (positive PCR test at the time of infection), and ongoing or newly expressed performance deficits lasting for at least 3 months prior to recruitment (minimum time since acute infection ≥90 days). This study only included PCS patients who did not suffer from post-exertional malaise and potential symptom exacerbations during rehabilitation were prevented through the use of a validated energy diary described elsewhere.^38^ In total, 12 PCS patients (7 female) between 40 and 63 years of age were included (Table 1, PCS). Performance deficits were documented according to the recent consensus statement,^6^ with the cluster of lead symptoms including fatigue/exercise intolerance, shortness of breath, and cognitive dysfunction impairing activity of daily living and everyday functioning.^6^ Fatigue was quantified using the Multidimensional Fatigue Inventory,^39^ which provides an aggregated score and two subscales pertaining to physical and mental fatigue (scores range from 0 to 100, with higher values indicating elevated levels of fatigue). The MoCA test^40^ was used to assess cognitive function (Table 1). A detailed clinical workup was performed, and history of comorbidities and current medication were documented. Further details can be found in the Results section.

**Table 1:** Demographic data of the groups

		PCS (*N* = 12)	CAD (*N* = 12)	CTRL (*N* = 12)
Sex		7 female/5 male	7 female/5 male	7 female/5 male
Age (years)	Mean ± SD^a^	55.1 ± 8.0	57.3 ± 6.2	26.7 ± 3.0
	Median/IQR^b^ [range]	57.6/10.1 [40.0, 63.7]	58.4/8.5 [47.8, 67.3]	27.4/5.1 [27.0, 30.8]
MFI-20^c^	Mean ± SD^a^	81.5 ± 8.6		
	Median/IQR^b^ [range]	79.5/13.5 [71, 99]		
MoCA^d^	Mean ± SD^a^	23.8 ± 6.1		
	Median/IQR^b^ [range]	25.0/6.3 [15, 28]		

^a^Standard deviation; ^b^Interquartile range; ^c^Multidimensional Fatigue Inventory, ^d^Montreal Cognitive Assessment.

#### CAD patients

A group of 12 patients (7 female) between 47 and 67 years of age with a diagnosis of CAD enrolled in a prospective cohort study on the effectiveness of medical rehabilitation was included for comparison (Table 1, CAD). CAD patients after acute myocardial infarction and/or reperfusion via percutaneous transluminal coronary angioplasty were included, matched for age and sex to the enrolled PCS patients. A detailed clinical workup including comorbidities, medication, and cardiopulmonary exercise testing was available for this group.

#### CTRL participants

A group of 12 young (self-reported) healthy participants (7 female) between 27 and 30 years of age were included as additional control group. Participants in the CTRL group were selected so that they had an age difference to the youngest patient (Table 1, PCS: 40.4 years) of at least ∼10 years. Furthermore, the last COVID infection should have been at least 10 months ago without PCS-specific symptoms. The demographic data of the groups are summarized in Table 1.

### Experiments

Several months after infection, patients with PCS still show hypometabolism in the dorsolateral prefrontal cortex,^15,16^ particularly in areas associated with executive function. To examine these areas more closely, a subset of two experimental cognitive tests from the cognitive Battery of the Berlin Aging Study^41^ was applied. As indicators for executive control, response inhibition and selective attention was measured by a modified version of the Flanker Task with three response conditions.^41^ Working memory was measured by a letter n-Back Task.^42,43^ The two tasks were programmed using Presentation software version 23.1 build 09.29.22 (Neurobehavioral Systems, Inc., Berkeley, CA, USA) as described elsewhere^44^ (cf. Supplement for a detailed description). They were presented on a Dell Latitude 3490 notebook (14-inch screen, Dell Technologies, Round Rock, TX, USA). Before the experiment started, all participants performed either one or two practice blocks per task (15–30 stimuli) to familiarize themselves with the task.

#### Flanker task

The Flanker task assesses selective attention and response inhibition and effects on Flanker performance have been reported following a COVID-19 infection.^45,46^ Participants had to indicate the colour of a central circle of a cross-shaped arrangement of five circles by pressing a key.^47^ The entire task consisted of seven blocks, each lasting 58.8 s, with a 30.6-s pause between each block, resulting in a total duration of 595.2 s. The central circle was centred on the middle of the screen and could had either red or green colours (response colour). The peripheral circles were green, red or blue, depending on the condition: congruent, incongruent or neutral. The three possible conditions were presented in a pseudo-randomized manner within a block of stimulations. A detailed description of the task and its graphical representation is given in the Supplementary material.

#### N-back task

The N-back was used to assess working memory and sustained attention since attention deficits have consistently been reported in PCS.^48^ The task consists of presenting letters and participants had to respond by button press whether the currently displayed letter matches the previous one. Based on preliminary studies, we chose the 1-back task for our experiments, meaning that participants had to compare the current letter with the immediately preceding one. The task consisted of 10 blocks, each lasting 45.2 s, with a 27.0-s pause between each block, totalling 695 s. Participants had to respond within 1500 ms using a key press. A detailed description of the task and its graphical representation is given in the Supplementary material.

#### fNIRS—setup and acquisition

Mobile fNIRS systems enable the measurement of blood flow changes in superficial cortical regions^49^ allowing to study neural activity in real-world settings and under more natural conditions compared to neuroimaging techniques such as fMRI. Movement artefacts had no effect on the measurements because haemodynamic signals can be measured during cognitive load and free body movements.^50-52^

A mobile NIRSport system (NIRStar v. 15.2, NIRx Medical Technologies, Glen Head, NY, USA) was used to record brain activity during task performance as described.^50^ The setup comprised 16 double-tip optodes with 8 light emitting sources and 8 detectors. Sources and detectors were positioned with a distance of ∼3 cm in accordance with the international 10–10 system,^53,54^ taking into account the specifications provided by Homan *et al*.^55^ Sources emitted infrared light impulses at 10.2 Hz (continuous wave fNIRS) at two wavelengths (760 and 850 nm). Sources were time-multiplexed to prevent crosstalk between channels. One detector was used for short-channel correction measurements. In total, the setup comprised 17 long-channels and 8 short-channels covering in a bi-hemispherical layout the dorsolateral prefrontal cortex. The positions of the channels are shown in Supplementary Fig. 1C as a black line between sensors (red marks) and detectors (blue marks) and are named by their sensor and detector. Four different cap sizes (54, 56, 58, 60 cm; NIRScap, EASYCAP GmbH, Herrsching, Germany) were used for various head circumferences. Optodes were covered from external light sources (screen, ambient light and other potential sources) by an opaque cover. Data were recorded using software Aurora fNIRS (Version 2023.9.3, NIRx Medical Technologies, Glen Head, NY, USA) on a Dell Latitude 3490 5410 notebook (Dell Technologies, Round Rock, TX, USA).

#### Stanford sleepiness scale

To evaluate the effectiveness of the cognitive tasks in terms of cognitive load, momentary sleepiness was measured using the Stanford Sleepiness Scale. The validated German version^56^ of the Stanford Sleepiness Scale (SSS)^57,58^ to gauge instantaneous sleepiness at the start of the measurements and after each task, using it as an indicator of cognitive load.^59^ Comprising just one item, the scale required respondents to select one of seven statements best representing their level of perceived sleepiness.^56,58^ As a single-item measure, the scale was particularly well-suited for repeated use throughout a research study or treatment intervention.^60^

#### Procedure

The experiments were conducted in a dimly lit room. Participants sat upright in a chair facing the screen at a self-chosen distance that allowed them to comfortably perceive the stimuli. The stimulation computer was connected to the fNIRS recording device via a LAN cable and sent event markers to the recorder through the Lab Streaming Layer environment.^61^ These event markers indicated the start and stop of each stimulation block, the type and timing of each stimulus (e.g. Flanker Task: congruent, incongruent or neutral stimulus, N-back task: letter), and the type and timing of each response key press. The tasks were performed in the following order. The first 10 participants of a group performed the Flanker-Task followed by a minimum five-minute break, after which they completed the N-back task. For the last two subjects of the group, the tasks were conducted in reverse order. The experiments involving PCS patients took place at times corresponding to peak alertness. Specifically, sessions were conducted between 10:30 and 12:00 and 13:00 and 14:30. The assessments of the CAD and CTRL groups were conducted between 9:30 and 17:30, depending on therapy schedules (CAD) or personal preferences and availability (CTRL).

### Data analysis

#### fNIRS measurement

A detailed description of the fNIRS measurement is given in the Supplementary material. In short for fNIRS data analysis, we used MATLAB with HOMER3 (v1.71.1),^62^ employing the Molavi and Dumont algorithm for motion correction.^63,64^ The data underwent band-pass filtering (0.05–0.005 Hz) and transformation into concentration changes of oxy- and deoxyhaemoglobin using the modified Beer−Lambert approach.^62^ To account for extracerebral contamination, a general linear model (GLM) was applied to regress out short-distance channel signals from the haemodynamic response function. To account for baseline drift, we used a third order polynomial fit. As implemented in this function, short-separation regression was performed with the short-separation channel, which showed the highest correlation with the respective long-separation channel.^65-67^ Individual measurements were baseline corrected (over 5 s before stimulus onset). For the subsequent variability analysis, the change of the HbO values (ΔHbO in µmol/l) for the entire duration of a task were exported, in total from 5 s prior to the start until 5 s after the final stimulation. Consequently, the total analysis period was 610 s for the Flanker task and 705 s for the N-back task.

#### Cognitive performance

In order to assess performance on both the Flanker and N-back tasks, two key performance indicators were analysed for each participant: accuracy and reaction time. The term ‘accuracy’ was defined as the ratio of all correct responses to the total number of answerable stimuli, irrespective of the type of stimulus presented. With regard to reaction time (RT), the following criteria were applied: (i) All responses with an RT < 100 ms were excluded as fast outlier. (ii) The mean RT and standard deviation (SD) were then calculated for each participant individually across all correct responses of the same stimulus type. RTs of a stimulus type that exceeded 2 SDs longer than its mean RT were excluded as a slow outlier. Finally, individual mean RTs were calculated as the average value of all remaining responses in milliseconds.

#### Variability analysis

To determine the regularity of a dynamic system and thereby identify the presence of recurring patterns in the time series, a family of statistics known as entropy is suitable.^32^ It has been shown that the sample entropy metric is effective in demonstrating differences between healthy controls and Alzheimer's disease in fNIRS signals.^68^ Sample entropy measures the regularity of time series, whereby the higher the value, the more irregular the data.^30,31^ Data analysis as well as statistical analysis was performed using the R 4.4.0 base package.^69^ To calculate the temporal variability, the sample entropy was calculated for each long-channel of a measurement and averaged over all 17 long-channels of a participant and task (Flanker Task: 6208 data points, N-back Task: 7173). The sample entropy analysis was performed using the function SampEn() from the R package TSEntropies^70^ with standard parameters (dim = 2, r = 0.2), which is an implementation of the original algorithm.^30,31^ The individual spatial variability during a task was calculated as the coefficient of variation (CV) of the sample entropy of all 17 long-channels (CV(SampEn)).

#### Statistical analysis

Group differences were calculated using ANOVA with one between-subjects factor (group, PCS, CAD, CTRL) via the R package ez.^71^ Effect size was given as partial eta-squared (η^2^) using R package apaTables^72^ and *post hoc* power calculation was done using R package pwr.^73^  *Post hoc* comparisons were based on Tukey’s honestly significant difference test (Tukey’s HSD), when appropriate.

## Results

PCS patients were referred to rehabilitation with an average age of 55.1 ± 8.0 years, and a mean time interval between first infection and start of medical rehabilitation of 314.3 ± 108.2 days. During the acute phase of the infection, all PCS patients received ambulant care or acute care at home, none required in-hospital care. Lead symptoms were self-reported and documented as part of the initial clinical examination. Cognitive dysfunction as a lead symptom was present in 75%, while fatigue/exercise intolerance and shortness of breath were observed in 83% and 100% of patients, respectively. Exercise intolerance was confirmed using cardiopulmonary exercise testing. Fatigue was objectively measured using MFI (Multidimensional Fatigue Inventory) scores (81.5 ± 8.2, Table 1: MFI-20), and self-reported cognitive dysfunction was assessed using the MoCA (Montreal Cognitive Assessment) (Table 1). In addition to PCS, patients were predominantly affected by circulatory and musculoskeletal disorders, as previously reported.^74^ Among the included patients, 75% had hypertension, 58% were obese, and 25% suffered from depression, corresponding to treatment with angiotensin II receptor blockers in 58%, and antidepressants in 33% of patients (Supplementary Table 1). The presence of vascular risk factors, including hypertension, diabetes, hyperlipidaemia, obesity, and smoking were comparable between PCS and CAD patients. Results of routine blood analyses were within the reference range (data not shown). Overall, 17% of PCS patients were active smoker.

### Task performance and sleepiness

**Figure 1:**
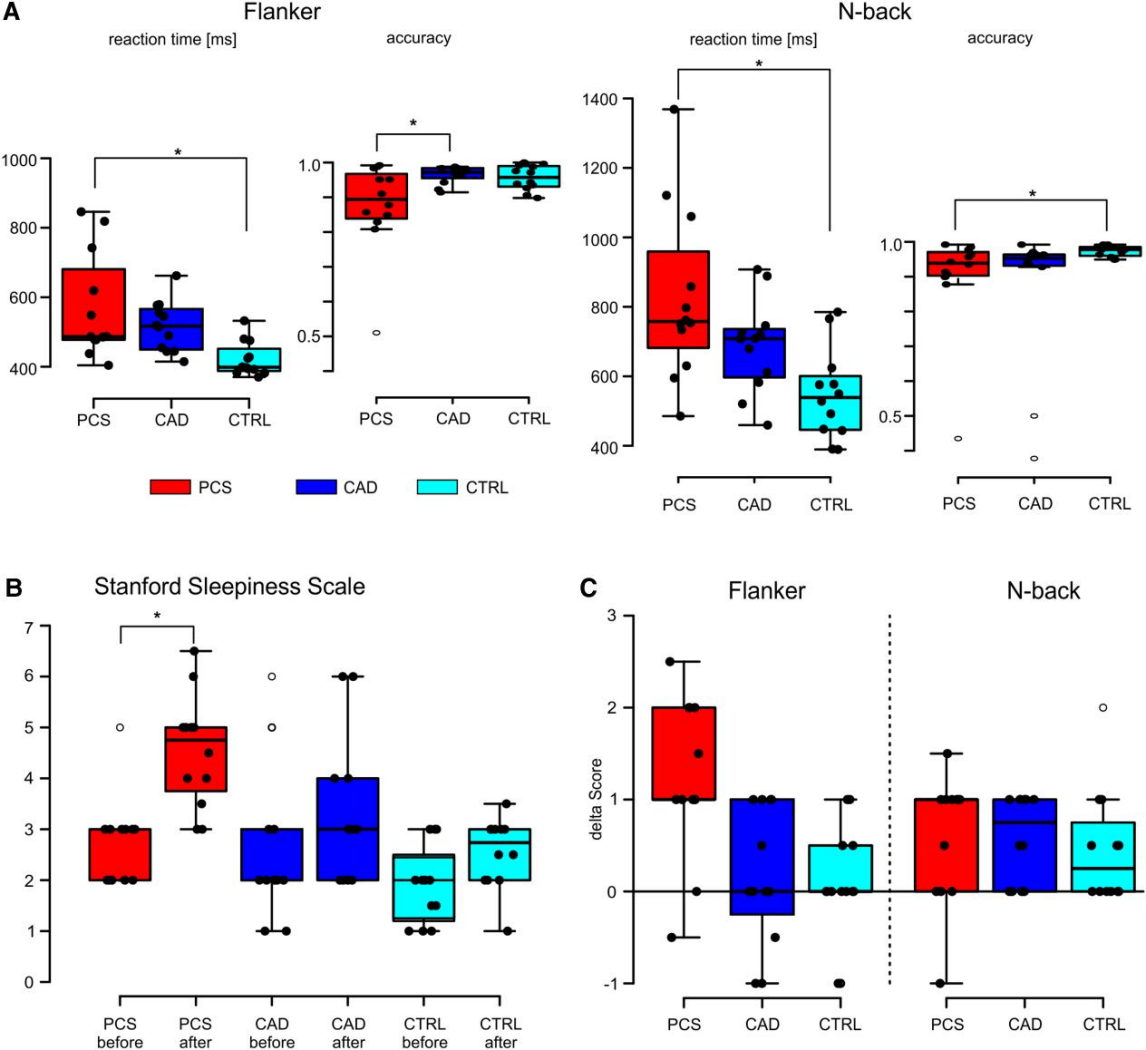
**Distribution of behavioural data and Stanford sleepiness scale scores in the flanker task and N-back task by group.** The boxplots represent (A) the distribution of the median reaction time, and the accuracy of task performance for the Flanker task (*left*) and N-back task (*right*), (B) the distribution of the Stanford Sleepiness Scale values before- and after the measurement, (C) the change in Stanford Sleepiness Scale score after completing a task compared to the situation preceding the task. In all graphs: post-COVID-19 syndrome (PCS) in red, coronary artery disease (CAD) in blue, and control (CTRL) in cyan. The boxes represent the mean 50th percentile, the thick line represents the median, and the whiskers the range of data belonging to each group. Outliers are represented as circles. Between-group comparison was conducted using ANOVA, * indicates significant difference between groups according to Tukey’s HSD (*P* < 0.05).

Since PCS patients show increased sleepiness during cognitive stress, the effectiveness of the cognitive tasks in terms of cognitive load was assessed using the Stanford Sleepiness Scale. Individual performance in the cognitive tasks was assessed through reaction time and accuracy (Table 2). In both cognitive tasks (Flanker and N-back), a significant group difference was observed in reaction times and accuracy rates for both tasks (ANOVA, *P* < 0.05). The PCS group demonstrated significantly longer reaction times than the control group in both tasks (Tukey’s HSD, *P* < 0.05), whereas CAD patients had comparable reaction times as CTRL in both tasks (Fig. 1A). With respect to accuracy rates, the PCS group performed significantly worse than the CAD group in the Flanker task (Tukey’s HSD, *P* < 0.05) and significantly worse than CTRL in the N-back task (Tukey’s HSD, *P* < 0.05). CAD patients displayed accuracy rates intermediate between those of PCS and CTRL (Fig. 1A, see Table 2).

**Table 2:** Results of the ANOVA and *post hoc* tests

Parameter	Task	Group	Mean ± SD^a^	*F* (DoFs)^b^	*P*	*η^2^*	*Post hoc* Power	Tukey’s HSD^c^	
								−CAD	−CTRL
Sample entropy	Flanker	PCS	0.024 ± 0.005	6.21 (2, 33)	0.005	0.27	0.734	−0.005	0.000
		CAD	0.029 ± 0.004						0.005
		CTRL	0.024 ± 0.005						
	N-back	PCS	0.026 ± 0.005	3.89 (2, 33)	0.030	0.19	0.560	−0.005	−0.001
		CAD	0.030 ± 0.003						0.003
		CTRL	0.027 ± 0.004						
CV (SampEn)^d^	Flanker	PCS	0.253 ± 0.090	2.45 (2, 33)	0.102				
		CAD	0.197 ± 0.064						
		CTRL	0.254 ± 0.059						
	N-back	PCS	0.263 ± 0.055	7.61 (2, 33)	0.002	0.32	0.926	0.050	0.091
		CAD	0.213 ± 0.069						0.042
		CTRL	0.172 ± 0.045						
Reaction time^e^	Flanker	PCS	544.0 ± 130.4	5.52 (2, 30)	0.009	0.27	0.828	37.6	122.4
		CAD	506.5 ± 74.5						84.9
		CTRL	421.6 ± 49.5						
	N-back	PCS	775.8 ± 186.7	7.33 (2, 30)	0.003	0.33	0.931	130.8	229.6
		CAD	645.0 ± 98.1						98.8
		CTRL	546.3 ± 129.8						
Accuracy	Flanker	PCS	0.91 ± 0.07	4.06 (2, 30)	0.028	0.21	0.681	−0.05	−0.05
		CAD	0.96 ± 0.03						0.00
		CTRL	0.96 ± 0.04						
	N-back	PCS	0.94 ± 0.04	4.56 (2, 30)	0.019	0.23	0.613	−0.01	−0.03
		CAD	0.96 ± 0.02						−0.02
		CTRL	0.97 ± 0.02						

^a^Standard deviation; ^b^DoFs, degrees of freedom; ^c^Tukey’s honestly significant difference test; ^d^Coefficient of variation of the sample entropy; ^e^Reaction time in [ms].

All groups had comparable sleepiness scores at median level and all three groups exhibited an increase in sleepiness throughout the experiment confirming cognitive stress (Fig. 1B). However, the most pronounced increase of 1.75 points (from 3 to 4.75, respectively *P* < 0.001) was observed in the PCS group, while the increases for the CAD and CTRL groups were only at trend level (*P* = 0.1), with increments of 1 point and 0.75 points, respectively (Fig. 1B). In detail, only the PCS group showed a significant increase in sleepiness after completing the Flanker task (Wilcoxon signed-rank test, *P* < 0.01; Fig. 1C, Flanker-PCS) while all groups demonstrated a significant increase in sleepiness, i.e. cognitive load, following the N-back task (*P* < 0.05; Fig. 1C).

### Variability of haemodynamic intermittence

**Figure 2:**
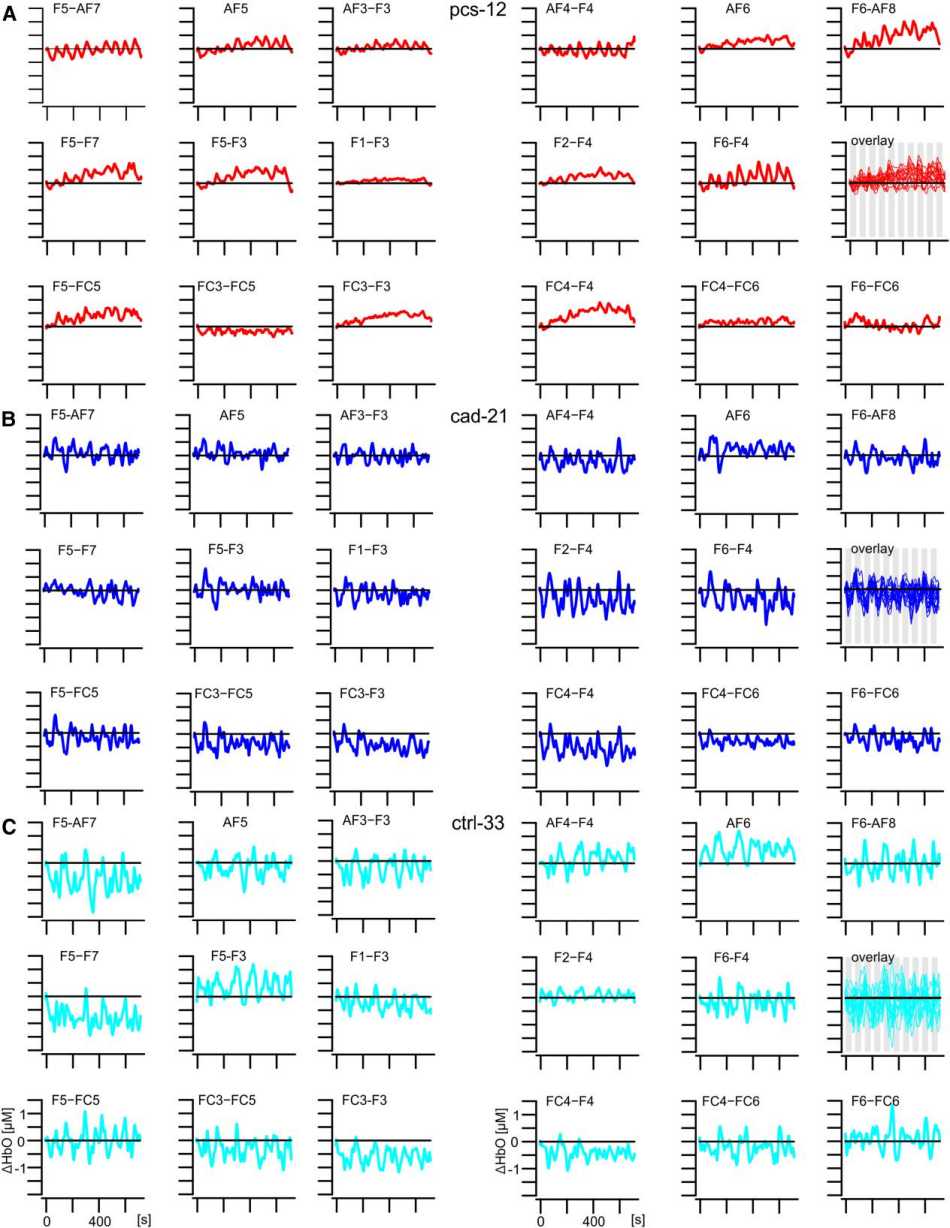
**Spatial representation of changes in oxygenated haemoglobin (ΔHbO) for three representative subjects during the N-back task.** (**A**) PCS patient pcs-12 in red. (**B**) CAD patient cad-21 in blue. (**C**) CTRL subject ctrl-33 in cyan. For all insets, the display covers the entire measurement period from 5 s before the start (baseline) to 700 s, horizontal black line indicates zero (i.e. no change). The label above each inset indicates the position of the channel *-*. The inset *overlay* (far right in the middle row) superimposes all 17 channels to visually represent the intra-individual spatial variability of the haemodynamic response. Spatial variability was calculated using the coefficient of variation of the sample entropy (CV(SampEn)) of all 17 channels. Grey vertical bars in the inset *overlay* mark the stimulation intervals.

The temporal dynamics of cerebral blood flow while performing cognitive tasks were assessed using fNIRS, and the variability was determined by calculating the sample entropy of these temporal changes for all 17 channels. As the N-back task induced changes in sleepiness score (Fig. 1C, N-back), characteristic patterns of haemodynamic responses during the N-back task were illustrated for this task in Fig. 2, using three representative participants (Fig. 2; pcs-13, cad-21 and ctrl-33, respectively). Notably, both the CAD patient and the CTRL participant displayed continuous fluctuations in blood flow over the entire prefrontal cortex. These fluctuations occurred independently of cognitive stimulation (Fig. 2B and C inset: overlay, the grey vertical bars indicating the timing of stimulation) and exhibited high temporal variability of cerebral blood flow across all channels in both subjects (mean sample entropy: cad-21 = 0.031, ctrl-33 = 0.028), accompanied by low spatial inter-channel variability within each subject (CV(SampEn): cad-21 = 0.099, ctrl-33 = 0.084). For comparison: A fluctuation in blood flow directly following the stimulation sequence used in the experiment, which consisted of ten blocks with pauses between them, would result in a sample entropy value of only 0.006. A significantly different pattern was observed in the PCS patient (Fig. 2A, pcs-12) and only a few channels exhibited similar behaviour to that seen in the two control subjects (e.g. Fig. 2A: inset F5-AF7, sample entropy = 0.022). Moreover, the majority of channels either showed almost no response throughout the task duration (e.g. Fig. 2A: inset F1-F3, sample entropy = 0.015) or a gradual increase in perfusion over the course of the task (e.g. Fig. 2A: inset FC4-F4, sample entropy = 0.010). Consequently, this PCS patient exhibited lower mean temporal variability of cerebral blood flow (mean sample entropy: pcs-12 = 0.019) and higher spatial inter-channel variability within themselves (CV(SampEn): pcs-12 = 0.355). The findings indicated an overall impairment in the intermittent regulation of cerebral blood flow.

**Figure 3:**
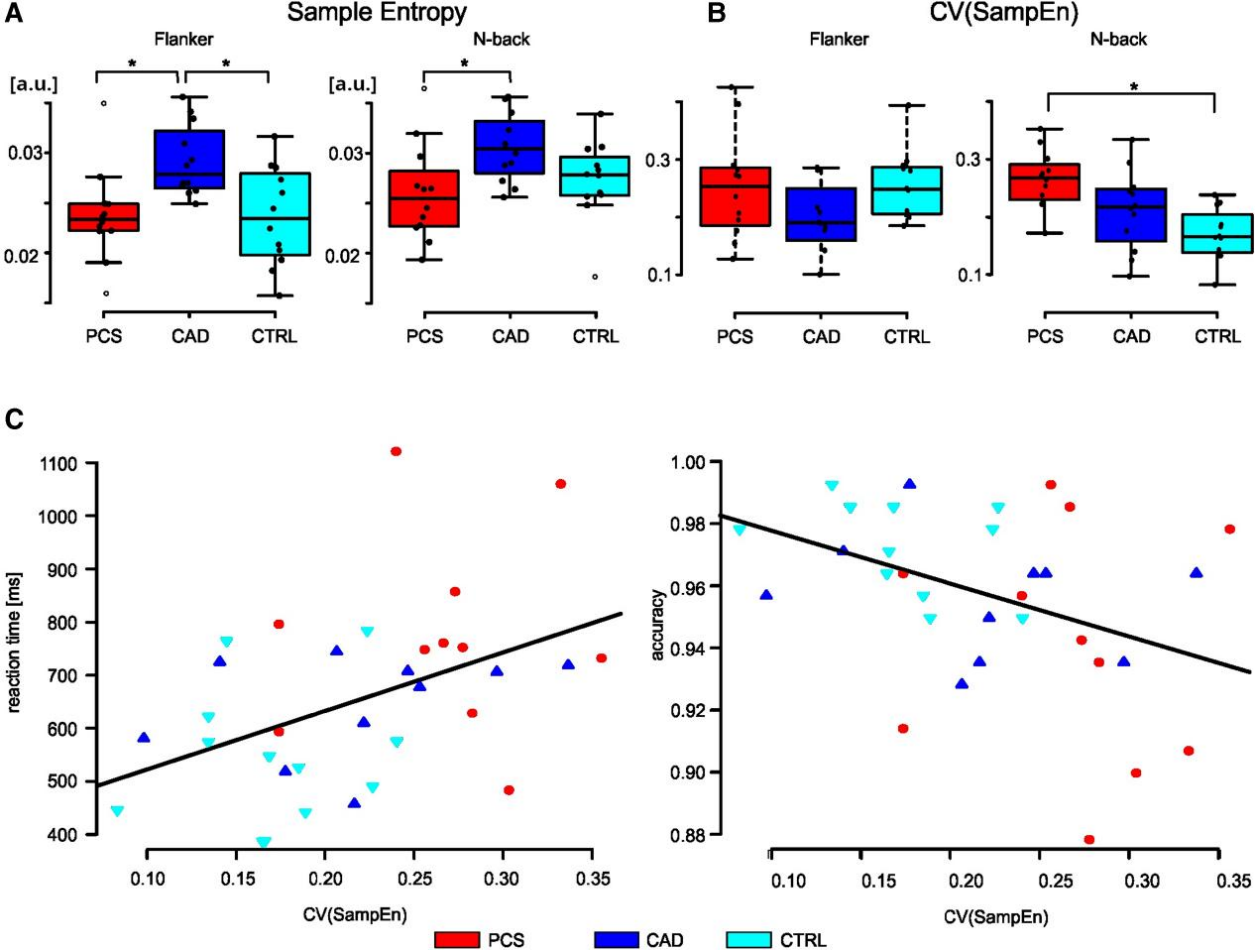
**Temporal and spatial variability of blood flow during the flanker task and N-back task and association of spatial variability and N-back task reaction time and accuracy.** The boxplots represent (**A**) the distribution of the temporal variability indicating the intermittency of hemodynamic changes as mean sample entropy in arbitrary units and (**B**) the distribution of the spatial variability of intermittency (CV(SampEn)) for both tasks. **A** and **B**, PCS in red, CAD in blue, and CTRL in cyan. Between-group comparison was conducted using ANOVA, * indicates significant difference between groups according to Tukey’s HSD (*P* < 0.05). (**C**) Regression analyses of spatial variability of intermittency (CV(SampEn)) in the N-back task and mean reaction time (*left*) and task accuracy (*right*). The data points show the individual data as follows: PCS, red filled circle; CAD, blue filled triangle point-up; CTRL, cyan-filled triangle point down.

In terms of overall task-specific differences, analysis of variance (ANOVA) revealed significant differences between the groups in terms of temporal variability (see Table 2, sample entropy, Fig. 3A) and subsequent *post hoc* analyses showed significant disparities between PCS and CAD in both tasks (see Table 2: Tukey's HSD; Fig. 3A). Furthermore, a significant difference was demonstrated between the CAD and CTRL group in the Flanker task (see Table 2: Tukey's HSD; Fig. 3A). To examine the effect of sex and age on sample entropy values, regression analyses were performed. The results showed that neither sex nor the interaction between sex and age had any influence on the sample entropy values. A significant association of age was only observed in the Flanker test for PCS (*P* = 0.025, uncorrected), where sample entropy decreased with increasing age. However, after applying the Bonferroni correction, the result was no longer significant. When analysing the task-specific individual spatial variability of blood flow in brain regions (see Table 2: CV(SampEn); Fig. 3B), there were no significant differences between the groups during the Flanker task (see Table 2: CV(SampEn), Flanker; Fig. 3B). During the N-back task, however, the spatial variability differed between groups, in that it decreased from PCS to CAD to CTRL (see Table 2: CV(SampEn), N-back). Subsequent *post hoc* analyses indicated that these differences were statistically significant between PCS and CTRL (Table 2: CV(SampEn), N-back; Fig. 3B). Overall, the combination of temporal and spatial variability (sampling entropy and CV(SampEn)) enabled distinction between groups based on fluctuations in blood flow regulation indicating that there are distinct regulatory mechanisms affected in PCS patients.

To assess whether spatial variability correlated with poorer performance in terms of longer reaction times and lower accuracy, regression analysis investigating relationships between the performance values-reaction time and accuracy-in the N-back task and the individual variation in spatial variability (CV(SampEn)). This analysis yielded a significant regression for both parameters (Table 3 reaction time and accuracy, respectively; Fig. 3C). Increasing coefficient of variation correlated with longer reaction times and reduced accuracy rates.

**Table 3:** Regression analysis

	Reaction time	Accuracy
Intercept	412.1 ± 91.3	0.996 ± 0.016
CV (SampEn)^a^	1102.5 ± 399.8	−0.172 ± 0.069
Adjusted *R^2^*	0.17	0.14
*F(*DoFs)^b^	7.60 (1, 31)	6.17 (1, 31)
*P*	0.01	0.02

^a^Coefficient of variation of the sample entropy; ^b^DoFs degrees of freedom.

## Discussion

In this study, the blood flow regulation of the dorsolateral prefrontal cortex was quantitatively analysed in PCS patients and two control groups while performing two different cognitive tasks using fNIRS. We hypothesized that specific differences in the signal structure of blood flow regulation between PCS patients and age-matched controls could be observed, and that these two groups differ from younger healthy controls. Further we assumed that spatial variability in more severely affected areas leading to decreased cognitive performance in PCS patients could be detected. In brief, our main finding was that PCS patients displayed disturbed intermittency in blood flow regulation as indicated by a more rigid blood flow signal structure particularly during the N-back task, which targets working memory and sustained attention. In addition, a higher spatial variability compared to the other groups was seen in PCS patients, which correlated with poorer performance in terms of longer reaction times and lower accuracy, suggesting that cognitive impairment in PCS depended on the disturbed adaptation of regional cerebral blood flow.

In our setup, cognitive performance was measured using two consecutive tests, the Flanker task, a task of selective attention and response inhibition particularly demanding the frontal and parietal cortex, and the N-back task, a working memory task demanding particularly the dorsolateral prefrontal cortex.^45,48,75,76^ While the groups showed no differences in sleepiness before the experiment, sleepiness increased in the PCS group after each task, whereas an increase for the control groups was only observed after the N-back task.

In general, blood flow is regulated by the capillary pericytes together with the smooth muscle cells of the arteries and arterioles, which react to the local energy demand by increasing the capillary diameter. Changes in blood flow in arterioles and capillaries are therefore intermittent and variable over time, which can be determined as sample entropy. In terms of cerebral blood flow alterations, we observed that PCS patients showed a more rigid and likely disturbed intermittency in blood flow regulation indicated by altered sample entropy. The impaired blood flow regulation may be caused by endothelial dysfunction^8,77^ in the cortex, including the dorsolateral prefrontal cortex.^77^ Disruptions of the endothelium can lead to increased capillary transit time heterogeneity (CTH), reducing oxygen supply to tissues.^27,28^ To compensate, local blood flow increases through enhanced blood influx into the capillary network and a rise in shunt capillaries.^27,28^ However, endothelial changes caused by COVID-19 may temporarily elevate CTH, critically lowering tissue oxygen levels and potentially triggering symptoms like memory impairment or cognitive fatigue.^29^ The spatial distribution of the temporal parameter sample entropy indicates how uniformly a brain region responds to load or how differently it responds due to damage. Regional differences can thus be determined as the coefficient of variation of the sample entropy (CV(SampEn)) of all fNIRS long channels. In our series, differences in blood flow regulation were highest in PCS and lowest in CTRL during the N-back task, reflected in their spatial variability. Our results indicated that the more coherent the response of the cortex to the load, the better the performance. These observations are in line with findings from resting-state fMRI studies in PCS patients, suggesting that correlations between fatigue severity, cognitive performance and daytime sleepiness exist for several brain regions.^78^ Furthermore, other studies also revealed an association between neurological symptoms and cognitive performance (poorer attention and episodic memory) in PCS patients by use of multiple linear regression analyses.^24^ This was reflected in a resting-state fMRI study of the dorsolateral prefrontal cortex, which showed a positive correlation with areas involved in focused attention and working memory, and a negative correlation with regions that are routinely deactivated during cognitive tasks that require attention.^79^ The latter includes the default mode network,^80,81^ which is believed to play a role in reflecting on thoughts and memories. The dorsolateral prefrontal cortex's almost constant activity in PCS patients, even during pauses between stimulation blocks (see Fig. 2A), suggests that these patients lack the opportunity for necessary self-reflection. This can lead to fatigue and general exhaustion during cognitive tasks. However, cognitive symptoms in PCS are heterogeneous; some domains improve over time, but many patients show persistent deficits, and brief screening tests may underestimate subjective impairments.

A recent fMRI study has shown that reduced oxygenation of the total deep grey matter (including the brainstem) was associated with an increased risk of excessive daytime sleepiness in PCS.^21^ This observation may at least partly be explained by endothelial dysfunction and a lasting reduction in function and density of small capillaries in PCS.^18,19^ Loss of these capillaries may lead to a significant reduction in grey matter volume in cortical and limbic areas of PCS patients.^24,25,77^ In addition, reduced capillary density may cause an undersupply of the affected brain tissue,^26^ which at the same time has a higher metabolic demand. This structural change may result in a restriction of intermittent blood flow regulation^82^ as observed in our study. It has been shown that during a SARS-CoV-2 infection, pericyte-mediated capillary constriction occurs, thereby reducing cerebral cortical blood flow by ∼17% leading to attentional deficits.^12^ Consequently, regulation becomes more rigid (i.e. lower sample entropy) and less able to adapt to local demand. These finding align with recent research demonstrating that oxygen supplementation via portable devices improved global cognition, attention, visual spatial/executive function and reduced depressive symptoms in PCS patients.^83^ It is also noteworthy that structural changes in the brain correlate with functional changes.^24^ Thus, hypometabolism has been found in patients with PCS in both the dorsolateral prefrontal cortex and the orbitofrontal, parietal, temporal and limbic cortices,^84^ as well as hypoconnectivity of the para-hippocampus and orbitofrontal regions.^25^ An EEG study demonstrated that brain activity in PCS patients at rest is decreased in the prefrontal and temporal cortices and that the functional brain network has undergone reorganization.^85^ Notably, the functional connectivity density of the dorsolateral prefrontal cortex has been found to mediate the relationship between grit and motivation in PCS patients.^86^ Consequently, it is crucial to reassess, the notion that psychosomatic factors are causally present in these patients^4,87^ should be reconsidered.

### Limitations

It should be noted that fNIRS measures changes in blood flow relative to reference time (here: 5 s before stimulation). In brain areas where the endothelium is damaged, increased CTH may occur in combination with a reduction in tissue oxygen supply.^27-29^ Compensation for the oxygen deficiency might cause increasing blood flow in the microcirculation.^27,28^ This generally leads to an increase in oxyhaemoglobin in the outflowing blood^29^ and would thus lead to a shift in the baseline reference value of the fNIRS measurement in PCS relative to CAD or CTRL. However, due to the nonlinearity between the fNIRS optical density measurement and the actual oxyhaemoglobin content e.g.,^88,89^ an increase in oxyhaemoglobin concentration may be underestimated. Since sample entropy reflects irregular blood flow control rather than the magnitude of flow changes, this aspect would only have limited effects on our findings. With increased neural load, however, additional capillary dilation may not be adequately performed as required, since the contractile pericytes surrounding the capillaries do not relax sufficiently post infection.^12^ This may be indicated by weak and delayed blood flow changes in PCS and lower sample entropy in PCS compared to CAD. Of note, in individual PCS patients, some channels exhibited near-normal intermittency with high sample entropy, whereas other channels demonstrated minimal change. This discrepancy manifested as elevated CV(SampEn) in PCS in comparison to CAD and CTRL. Consequently, the individual CV(SampEn) might be regarded as an indicator of the regional manifestation of altered blood flow. This was corroborated by the observation that CV(SampEn) correlated with cognitive task performance. Another limitation of the study may be that the order of the tests was non-randomized and the increased sleepiness in the N-back task could have been caused by the preceding Flanker task. However, reaction times and accuracies in the N-back task did not differ significantly, regardless of the order. It is worth mentioning that the results presented here were collected in PCS patients able to participate in an (exercise-based) medical rehabilitation and may thus not be transferable to patients with less or more severe symptoms.

## Conclusion

This study showed that cognitive performance deficits in PCS patients correlated with disturbances in cerebral blood flow regulation in the microcirculation of capillary networks. We detected alterations in blood flow regulation in PCS based on variability measures which may represent an alternative method particularly given the inability to directly measure cortical structural changes of the microvasculature using fNIRS. Since altered cortical transit time was detected, we conclude that local and regional regulation of cortical blood flow is impaired in PCS. Our approach is therefore suitable and promising for assessing alterations of cortical blood flow regulation in PCS. It may also be applied as a sensitive method to detect the effects of (pharmacological) interventions in PCS or patients who share comparable pathophysiological mechanisms (e.g. ME/CFS^35,36^). Understanding altered flow patterns could illuminate not just the mechanisms underlying cognitive dysfunction in PCS, but the fundamental principles by which vascular health shapes long-term brain function.

## Supplementary Material

fcag036_Supplementary_Data

## Data Availability

The data that support the findings of this study are available from the corresponding author on reasonable request.
