# Supplementary material for: Assessment of dynamic cerebral blood flow changes during cognitive tasks in patients with post-COVID-19 syndrome
Source: Brain Commun. 2026 Feb 10;8(1):fcag036. doi: 10.1093/braincomms/fcag036 (PMC12917544; doi:10.1093/braincomms/fcag036)
Supplement: fcag036_Supplementary_Data [file fcag036_supplementary_data.pdf]

# Assessment of dynamic cerebral blood flow changes during cognitive tasks in patients with Post-COVID-19 Syndrome

Dieter F. Kutz, René Garbsch, Frank C Mooren, Boris Schmitz, & Claudia Voelcker-Rehage

# Supplementary Materials

# Methods

## Flanker Task

The Flanker task assesses selective attention and response inhibition and effects on Flanker performance have been reported following COVID-19 infection[1,2]. Participants had to indicate the colour of a central circle of a cross-shaped arrangement of five circles by pressing a key[3]. The entire task consisted of seven blocks, each lasting 58.8 s, with a 30.6-s pause between each block, resulting in a total duration of 595.2 s. Each block comprised 35 stimuli and started with the presentation of a white fixation cross on a black screen for 300 ms, followed by the presentation of the stimuli at intervals of 1000-1300 ms. A cross-shaped stimulus, with a diameter of 0.5 cm for each circle and a centre-to-centre distance of 1 cm between the outer circles and the central circle, was presented for a duration of 200 ms. This was then followed by a blank black screen. The central circle was centred on the middle of the screen and could had either red or green colours (response colour). The peripheral circles were green, red or blue, depending on the condition: congruent, incongruent, or neutral. Specifically, in the case of congruent condition, the central and peripheral circles exhibited the same colour (red or green), while for the incongruent condition, the peripheral circles displayed the opposite response colour to the central circle (e.g. red for the central circle and green for the peripheral circle, or green for the central circle and red for the peripheral circle). For the neutral condition, the peripheral circles were blue, irrespective of the response colour exhibited by the central circle. The three possible conditions were presented in a pseudo-randomised manner within a block of stimulations. A graphical representation of the possible combinations is shown in Fig. 1A. Participants were required to respond within 800 ms by pressing the <Down arrow> key (marked green) for green and <Right arrow> key (marked red) for red. There were six possible combinations of stimuli, three of which required responses corresponding to green (Fig. 1A, upper row) and three requiring responses corresponding to red (Fig. 1A, lower row).

## N-back Task

The N-back was used to assesses working memory and sustained attention since attention deficits have consistently been reported in PCS [4]. The task consists of presenting letters and participants had to respond by button press whether the currently displayed letter matches the previous one. Based on preliminary studies, we chose the 1-back task for our experiments, meaning that participants had to compare the current letter with the immediately preceding one. The task consisted of 10 blocks, each lasting 45.2 s, with a 27.0-s pause between each block, totalling 695 s. Each block comprised 15 presentations of a single letter for 500 ms at intervals of 2800-3200 ms. Participants had to respond within 1500 ms using a key press. If there was a match, they pressed the <Down arrow> key (marked green), and if not, they pressed the <Right arrow> key (marked red). Letters were presented centrally on the screen against a black background, measuring 2.7 cm horizontally and vertically. A theoretical sequence of the first five stimuli and correct answers is illustrated in Fig. 1B.

## Data Analysis

## fNIRS Measurement

fNIRS data analysis was performed in MATLAB (Math-Works, Natick, MA, USA) using HOMER3 (version 1.71.1)[5]. We used the algorithm described by Molavi and Dumont[6] as implemented in the HOMER3 hmrR_ MotionCorrectWavelet filtering function (inter-quartile range: 1.22[7]). Following motion-artifact correction, the data was band-pass filtered using 0.050 Hz as low pass cutoff frequency and 0.005 Hz as high pass cutoff frequency. Attenuation changes of both wavelengths (850 nm and 760 nm) were transformed to concentration changes of oxy- and deoxygenated hemoglobin (HbO and HbR, respectively) using the modified Beer-Lambert approach (partial pathlength factor: 6.0[5]). In order to regress extra-cerebral contaminations (measured by short-distance channels) out of the signal, we modelled the hemodynamic response function (HRF) by using a general linear model approach (GLM) that uses ordinary least squares and a consecutive sequence of Gaussian functions with a standard deviation of 0.5 s and their means separated by 0.5 s over a specific regression time (used parameters in HOMER3 hrmR_GLM function: glmSolveMethod 1; idxBasis 1; paramBasis 0.5 and 0.5). The parameter range depended on the task and was set to [-5, 605] for the Flanker Task and [-5, 700] for the N-back task. Furthermore, to account for baseline drift, we used a third order polynomial fit. As implemented in this function, short-separation regression was performed with the short-separation channel, which showed the highest correlation with the

respective long-separation channel[8-10]. Individual measurements were baseline corrected (over 5 s before stimulus onset). For the subsequent variability analysis, the change of the HbO values ($\Delta$HbO in µmol/l) for the entire duration of a task were exported, in total from 5 s prior to the start until 5 s after the final stimulation. Consequently, the total analysis period was 610 s for the Flanker task and 705 s for the N-back task.

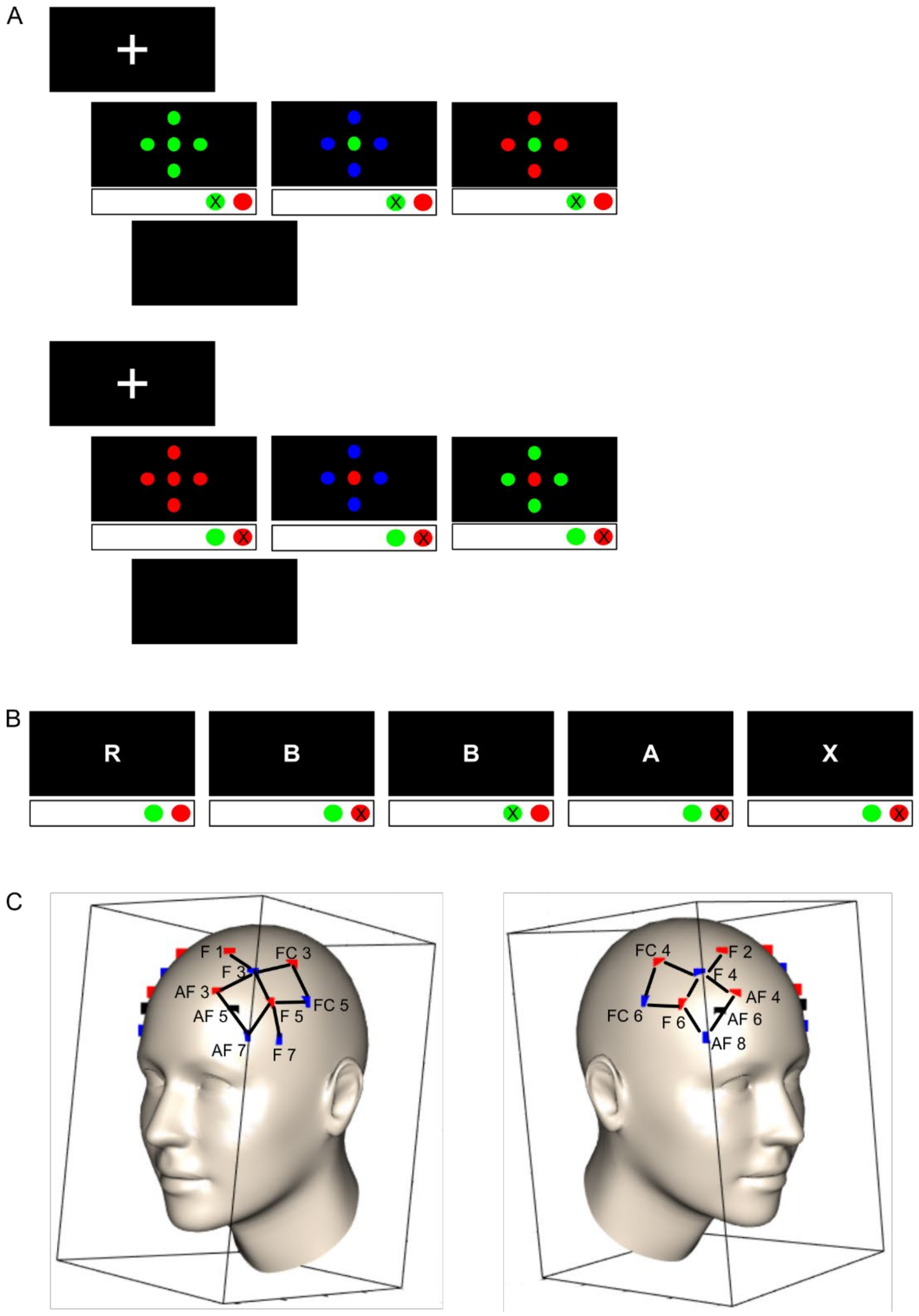

**Supplemental Figure 1: Illustration of tasks and channel distribution.** (**A**): Flanker Task - selective attention and response inhibition: The sequence and six different stimulus configurations are shown. The upper row displays the three stimuli that require pressing the green-marked key, while the lower row shows those requiring the red-marked key. In both rows: sequence from left to right is congruent, neutral and incongruent. (**B**) N-back task - working memory and attention: The theoretical sequence of the first five stimuli of the 1-back version and the correct answers are illustrated from left to right. When the displayed letter matches the previous one, participants are required to press the green button (e.g., middle inset); otherwise, they press the red button. (**C**) Distribution of the fNIRS channels: Channels are shown as black line between a sensor (red marks) and a detector (blue marks). The naming follows the international 10-10 system[11] with the principle <*sensor name*>-<*detector name*>. For example, the channel FC3–FC5 describes the brain area between the sensor FC3 and the detector FC5 (Fig. 1C, most posterior channel of the left hemisphere). Only for the channels comprising combinations of AF3–AF7 and AF4–AF8, respectively, were the names AF5 and AF6 employed, as these best describe the positions.

**Supplementary Table 1 Comorbidities and Medication of PCS and CAD patients**

| | PCS (N=12) | CAD (N=12) | *P* |
|---|---|---|---|
| **Comorbidities** | | | |
| Diseases of the circulatory system | 11 (92) | 12 (100) | 1.000 |
| *Arterial hypertension* | 9 (75) | 9 (75) | 1.000 |
| *Coronary artery disease* | 2 (18) | 12 (100) | **0.001** |
| *Myocardial infarction* # | 0 (0) | 10 (83) | **0.001** |
| Endocrine, nutritional or metabolic diseases | 9 (75) | 11 (92) | 0.590 |
| *Obesity* | 7 (58) | 5 (42) | 0.684 |
| *Hyperlipidemia* | 3 (25) | 5 (42) | 0.667 |
| *Diabetes mellitus* | 1 (8) | 1 (8) | 1.000 |
| *Hypothyroidism* | 2 (17) | 0 (0) | 0.478 |
| Mental and behavioral disorders | 7 (58) | 4 (33) | 0.414 |
| *Depressive/ adjustment disorders* | 3 (25) | 2 (17) | 1.000 |
| Diseases of the nervous system | 5 (42) | 2 (17) | 0.371 |
| Diseases of the respiratory system | 4 (33) | 2 (17) | 0.640 |
| Diseases of the musculoskeletal system and connective tissue | 4 (33) | 1 (8) | 0.317 |
| **Medication*** | | | |
| Beta blocker | 4 (33) | 12 (100) | **0.001** |
| Statin | 4 (33) | 11 (92) | **0.009** |
| AT-II receptor blocker | 7 (58) | 7 (58) | 1.000 |
| Anticoagulant | 4 (33) | 9 (75) | 0.100 |
| Analgesic | 5 (42) | 3 (25) | 0.667 |
| ACE inhibitor | 2 (17) | 4 (33) | 0.640 |
| Diuretic | 2 (17) | 4 (33) | 0.640 |
| Antidepressant | 4 (33) | 1 (8) | 0.317 |
| Calcium channel blocker | 2 (17) | 2 (17) | 1.000 |
| Glucocorticoid | 2 (17) | 1 (8) | 1.000 |
| Diabetes medication | 1 (8) | 0 (0) | 1.000 |

Data is presented as n (%). Between-group comparison was performed using Chi-square test. Diseases of the circulatory system (ICD-10 I00-I99); Endocrine, nutritional or metabolic diseases (ICD-10 E00-E90); Mental and behavioral disorders (ICD-10 F00-F99); Diseases of the nervous system (ICD-10 G00-G99); Diseases of the respiratory system (ICD-10 J00-J99); Diseases of the musculoskeletal system and connective tissue (ICD-10 M00-M99). # Primary indication for rehabilitation in CAD patients. * Medication at admission. For the majority of patients, medication remained unchanged.

## Supplementary References


1. Fassbender C, Foxe JJ, Garavan H. Mapping the functional anatomy of task preparation: Priming task-appropriate brain networks. *Human Brain Mapping*. 2006;27(10):819-827.
2. Hall PA, Ayaz H, Meng G, et al. Neurocognitive and psychiatric symptoms following infection with COVID-19: Evidence from laboratory and population studies. *Brain, Behavior, & Immunity - Health*. 2023;28:100595.
3. Voelcker-Rehage C, Godde B, Staudinger UM. Physical and motor fitness are both related to cognition in old age. *European Journal of Neuroscience*. 2010;31(1):167-176.
4. James GA, Hazaroglu O, Bush KA. A human brain atlas derived via n-cut parcellation of resting-state and task-based fMRI data. *Magnetic Resonance Imaging*. 2016;34(2):209-218.
5. Huppert TJ, Diamond SG, Franceschini MA, Boas DA. HomER: a review of time-series analysis methods for near-infrared spectroscopy of the brain. *Appl Opt*. 2009;48(10):D280-D298.
6. Molavi B, Dumont GA. Wavelet-based motion artifact removal for functional near-infrared spectroscopy. *Physiological Measurement*. 2012;33(2):259.
7. Carius D, Herold F, Clauß M, et al. Increased Cortical Activity in Novices Compared to Experts During Table Tennis: A Whole-Brain fNIRS Study Using Threshold-Free Cluster Enhancement Analysis. *Brain Topography*. 2023.
8. von Lühmann A, Ortega-Martinez A, Boas DA, Yücel MA. Using the General Linear Model to Improve Performance in fNIRS Single Trial Analysis and Classification: A Perspective. *Frontiers in Human Neuroscience*. 2020;14.
9. Yücel M, Selb J, Aasted C, et al. Short separation regression improves statistical significance and better localizes the hemodynamic response obtained by near-infrared spectroscopy for tasks with differing autonomic responses. *Neurophotonics*. 2015;2(3):035005.
10. Yücel MA, von Lühmann A, Scholkmann F, et al. Best practices for fNIRS publications. *Neurophotonics*. 2021;8(1):34.
11. Nuwer MR, Comi G, Emerson R, et al. IFCN standards for digital recording of clinical EEG. *Electroencephalography and Clinical Neurophysiology*. 1998;106(3):259-261.
